# Outcomes of Transcatheter Aortic Valve Implantation in Patients with and without Diabetes Mellitus

**DOI:** 10.21470/1678-9741-2023-0088

**Published:** 2024-07-15

**Authors:** Hüseyin Ayhan, Murat Can Güney, Telat Keleş, Engin Bozkurt

**Affiliations:** 1 Department of Cardiology, Faculty of Medicine, University of Health Sciences, Sincan Education and Research Hospital, Ankara, Turkey; 2 Department of Cardiology, Faculty of Medicine, Atılım University, Medicana International Ankara Hospital, Ankara, Turkey.; 3 Department of Cardiology, Faculty of Medicine, Ankara Yıldırım Beyazıt University, Ankara City Hospital, Ankara, Turkey.; 4 Department of Cardiology, Medicana International Ankara Hospital, Ankara, Turkey.

**Keywords:** Transcatheter Aortic Valve Replacement, Glycated Hemoglobin, Aortic Valve Stenosis, Diabetes Mellitus, Acute Kidney Injury

## Abstract

**Introduction:**

Diabetes mellitus (DM) in patients undergoing cardiac transcatheter or surgical interventions usually is correlated with poor outcomes. Transcatheter aortic valve implantation (TAVI) has been developed as a therapy choice for inoperable, high-, or intermediate-risk surgical patients with severe aortic stenosis (AS).

**Objective:**

To evaluate the impact of DM and hemoglobin A1c (HbA1c) on outcomes and survival after TAVI.

**Methods:**

Five hundred and fifty-two symptomatic severe AS patients who underwent TAVI, of whom 164 (29.7%) had DM, were included in this retrospective study. Follow-up was performed after 30 days, six months, and annually.

**Results:**

The device success and risks of procedural-related complications were similar between patients with and without DM, except for acute kidney injury, which was more frequent in the DM group (2.4% *vs.* 0%, *P*=0.021). In-hospital and first-year mortality were similar between the groups (4.9% *vs.* 3.6%, *P*=0.490 and 15.0% *vs.* 11.2%, *P*=0.282, respectively). There was a statistical difference between HbA1c ≥ 6.5 and HbA1c ≤ 6.49 groups in total mortality (34.4% *vs.* 15.8%, *P*<0.001, respectively). The only independent predictors were Society of Thoracic Surgeons score (hazard ratio [HR] 1.28, 95% confidence interval [CI] 1.09-1.51; *P*=0.003) and HbA1c level ≥ 6.5 (HR 10.78, 95% CI 2.58-21.50; *P*=0.003) in multivariable logistic regression analysis.

**Conclusion:**

In this study, we conclude that DM was not correlated with an increased mortality risk or complication rates after TAVI. Also, it was shown that mortality was higher in patients with HbA1c ≥ 6.5, and it was an independent predictor for long-term mortality.

## INTRODUCTION

In recent studies and guidelines, transcatheter aortic valve implantation (TAVI) has been demonstrated to be feasible and efficient to treat symptomatic severe aortic stenosis (AS), irrespective of the baseline risk degree^[1,2,3,4]^. Diabetes mellitus (DM) in patients undergoing cardiac transcatheter or surgical interventions usually is correlated with poor outcomes^[5,6]^. There is contradictory and lacking knowledge about the outcomes of DM systematically used in risk scoring systems in TAVI patients^[7,8,9]^. Although there are various results in some studies, according to a meta-analysis with 16 studies and 13,253 patients in total, 30-day and one-year survival and 30-day major complications were detected at similar rates in the groups with and without DM^[10]^. However, since these studies and meta-analysis do not answer all questions on this subject, some studies try to clarify this issue today^[11,12,13,14]^. Also, our knowledge about the effect of hemoglobin A1c (HbA1c) in TAVI patients is even more limited^[15]^. Thus, we sought to evaluate the impact of DM and HbA1c on outcomes and survival after TAVI.

## METHODS

This was a retrospective cohort study that included patients who had TAVI for severe AS in our tertiary center from July 2011 to December 2019. All patients were symptomatic, with New York Heart Association class II-IV. AS was evaluated initially with transthoracic echocardiography followed by transesophageal echocardiography or electrocardiogram-gated, multi-slice computed tomography (MSCT). The eligibility of patients for TAVI was selected by a multidisciplinary heart team. TAVI outcomes, device success, and complications were recognized according to the Valve Academic Research Consortium (or VARC) 2 definitions^[16]^. The TAVI procedure at our institute has been previously defined in detail^[17]^. In brief, patients undergoing TAVI with a multidisciplinary heart team were evaluated with clinical and imaging resources. All patients underwent invasive coronary angiography to recognize coronary artery disease (CAD) before TAVI. The access route (transfemoral or trans-subclavian) for TAVI was chosen according to iliofemoral artery size, calcification, and tortuosity on MSCT. The procedures were performed under general anesthesia in the first 74 patients and under local anesthesia with sedation in the following patients. Four types of aortic valves were used: Edwards SAPIEN XT®, SAPIEN 3® valve (Edwards Lifesciences, Irvine, California, United States of America), Lotus™ valve system (Boston Scientific, Massachusetts, United States of America), and ACURATE Neo™ (Boston Scientific).

Clinical follow-up was performed following 30 days, six months, then annually. The patients’ vital situation was approved through the last clinical follow-up or by telephone calls. Institutional ethical committee approved the study (Date, No: March 2011-068) and the need for informed patients’ consent about the procedure was waived.

The diagnosis of DM was documented based on the patient’s history, previous medical records, using medications, and the current HbA1c levels. Blood samples for serum glucose and HbA1c levels were collected within the first 24 hours before TAVI. In the present study, we applied previously reported HbA1c levels cutoffs for defining no DM and DM (< 6.49% and ≥ 6.5%, respectively) to stratify the outcomes. Patients were classified into two groups according to their DM: DM group and no DM group. TAVI was performed in 552 consecutive patients and 164 (29.7%) DM patients according to the abovementioned definition or HbA1c levels.

### Statistical Analyses

All tests were two-sided, and a *P*-value < 0.05 was considered statistically significant. Data analyses were performed with IBM Corp. Released 2011, IBM SPSS Statistics for Windows, version 20.0, Armonk, NY: IBM Corp. Continuous variables are shown as the mean ± standard deviation and were compared using a *t*-test. Categorical variables are shown as absolute numbers with frequencies (%) and were analyzed using a Chi-square or Fisher’s exact test. Normality was checked with the Kolmogorov-Smirnov test. Time-associated events were evaluated using Kaplan-Meier methods. The logrank test was used to test the equality of survival distributions. Multivariate adjusted Cox proportional hazard models were fitted for all-cause mortality as the dependent variable and adjusted to variables previously associated with mortality after TAVI.

## RESULTS

A total of 552 all-comer patients underwent TAVI at our institution, their mean age was 77.6 ± 7.9 years, which had statistical difference between DM and no DM groups (74.9 ± 8.7 *vs.* 78.8 ± 7.3 years, *P*<0.001, respectively). The baseline characteristics of the study patients were shown in Table 1. Of the 552 patients, 164 (29.7%) had DM according to history, medications, and HbA1c levels. As expected, patients in the DM group had higher rates of CAD and its risk factors, such as hypertension (HT), hyperlipidemia (HL), history of myocardial infarction (MI), and percutaneous coronary intervention (PCI). Despite these, there was no statistical difference in risk scores, but they were numerically higher in the DM group. There was a statistical difference in the use of antiplatelets/anticoagulants before TAVI. The use of dual antiplatelet was higher in the DM group (5.6% *vs.* 2.6%, respectively), while the use of anticoagulants was higher in the no DM group (22.2% *vs.* 24.6%, respectively). In the DM group, aortic valve area (AVA) was statistically higher, while the common femoral artery (CFA) diameter was smaller (AVA 0.68 ± 0.16 cm^2^ *vs.* 0.66 ± 0.16 cm^2^; CFA 7.2 ± 1.2 cm *vs.* 7.7 ± 1.1 cm).

**Table 1:** Baseline characteristics and laboratory parameters.

Parameters	All	DM	No DM	P-value
	n=552	n=164	n=388	
Age (years)	77.6 ± 7.9	74.9 ± 8.7	78.8 ± 7.3	< 0.001
Female, n (%)	302 (54.7)	88 (53.7)	214 (55.2)	0.747
BMI (kg/m2)	27.7 ± 6.1	29.1 ± 4.8	27.1 ± 6.5	0.010
NYHA, n (%)				0.983
2	144 (26.1)	44 (26.8)	100 (25.8)	
3	313 (56.7)	92 (56.1)	221 (57.0)	
4	83 (14.6)	24 (15.0)	59 (15.2)	
Pulmonary edema	12 (2.2)	4 (2.4)	8 (2.1)	
HT, n (%)	458 (83.0)	152 (92.7)	306 (78.9)	<0.001
HL, n (%)	277 (50.2)	131 (79.9)	146 (37.6)	<0.001
CABG, n (%)	130 (23.6)	55 (33.5)	75 (19.4)	<0.001
Previous PCI, n (%)	115 (20.9)	45 (27.4)	70 (18.1)	0.014
Previous MI, n (%)	66 (12.0)	30 (18.3)	36 (9.3)	0.003
PAD, n (%)	43 (7.8)	18 (11.0)	25 (6.4)	0.069
AF, n (%)	192 (24.0)	34 (20.7)	98 (25.4)	0.242
Stroke, n (%)	33 (6.0)	12 (7.3)	21 (5.4)	0.388
Previous valve surgery, n (%)				0.170
Mitral	17 (3.1)	5 (3.0)	12 (3.1)	
Aorta	7 (1.3)	1 (0.6)	6 (1.5)	
Moderate to severe COPD, n (%)	234 (42.4)	79 (48.1)	155 (39.9)	0.246
Chronic kidney disease, n (%)				0.085
Stage 1	63 (11.7)	27 (16.7)	36 (9.5)	
Stage 2	258 (47.9)	68 (42.0)	190 (50.4)	
Stage 3a	111 (20.6)	31 (19.1)	80 (21.2)	
Stage 3b	85 (15.8)	27 (16.7)	58 (15.4)	
Stage 4	22 (4.1)	9 (5.6)	13 (3.4)	
Renal replacement therapy, n (%)	13 (2.4)	2 (1.2)	11 (2.8)	0.251
STS score (%)	6.0 ± 3.3	6.6 ± 3.7	5.8 ± 3.1	0.052
EuroSCORE II (%)	9.0 ± 5.7	9.9 ± 6.8	8.6 ± 5.2	0.065
Logistic EUROSCORE (%)	22.6 ± 14.7	23.5 ± 14.4	22.2 ± 14.9	0.596
CAD, n (%)				< 0.001
Normal	125 (31.8)	29 (17.7)	146 (37.8)	
Non-obstructive	241 (43.8)	88 (53.7)	153 (39.6)	
Obstructive	134 (24.4)	47 (28.7)	87 (22.5)	
Need for PCI, n (%)	69 (12.5)	19 (11.6)	50 (13.0)	0.658
Pre-antiplatelet/anticoagulation (%)				0.037
ASA or P2Y12	72.6	72.3	72.8	
ASA + P2Y12	3.5	5.6	2.6	
Warfarin	20.4	18.5	21.2	
DOAC	3.5	3.7	3.4	
Post-antiplatelet/anticoagulation (%)				0.991
ASA or P2Y12 alone	3.2	4.5	2.7	
ASA + P2Y12	67.8	67.9	67.8	
Warfarin alone	6.8	6.4	7.0	
ASA + warfarin	4.5	4.6	4.5	
ASA + warfarin + clopidogrel	5.1	4.5	5.4	
Warfarin + clopidogrel	5.5	6.4	5.1	
DOAC	5.7	4.5	6.3	
DOAC + clopidogrel	1.0	1.2	0.9	
DOAC + ASA + clopidogrel	0.4	-	0.6	
Laboratory parameters				
Serum glucose (mg/dl)	127.4 ± 54.3	168.5 ± 72.3	109.9 ± 31.1	< 0.001
HbA1c %	6.30 ± 1.25	7.18 ± 1.38	5.76 ± 0.76	< 0.001
Total cholesterol (mg/dl)	168.9 ± 44.3	165.0 ± 47.4	170.5 ± 42.9	0.191
Triglyceride (mg/dl)	121.5 ± 63.9	132.3 ± 69.1	116.9 ± 61.0	0.010
LDL cholesterol (mg/dl)	100.2 ± 36.1	97.7 ± 39.2	101.3 ± 34.7	0.292
HDL cholesterol (mg/dl)	45.0 ± 13.6	41.5 ± 12.1	46.4 ± 13.9	< 0.001
Creatinine (mg/dl)	1.06 ± 0.52	1.09 ± 0.48	1.04 ± 0.53	0.277
Hemoglobin (mg/dl)	11.6 ± 1.9	11.5 ± 1.8	11.7 ± 1.9	0.431
Platelet count (× 103/L)	240.1 ± 82.8	255.3 ± 79.9	233.7 ± 83.3	0.005
Troponin (pg/ml)	84.6 ± 113.5	82.7 ± 122.6	85.1 ± 111.3	0.896
CK-MB (ng/ml)	4.4 ± 11.0	3.5 ± 3.6	4.6 ± 12.3	0.493
CRP (mg/dl)	7.2 ± 10.0	8.4 ± 13.6	6.7 ± 8.5	0.302
Baseline echocardiographic and MSCT parameters				
LVEF (%)	51.7 ± 14.0	50.4 ± 14.8	52.3±13.6	0.076
LVEDD (cm)	4.74 ± 0.66	4.81 ± 0.65	4.71±0.66	0.120
LVESD (cm)	3.14 ± 0.84	3.24 ± 0.87	3.10±0.83	0.082
LA (cm)	4.67 ± 0.65	4.66 ± 0.59	4.68±0.57	0.721
Aortic velocity (cm/s)	4.4 ± 0.61	4.4 ± 0.61	4.5±0.61	0.330
Aortic max gradient (mmHg)	82.0 ± 23.0	80.2 ± 21.8	82.8±23.5	0.222
Aortic mean gradient (mm Hg)	50.5 ± 15.1	49.1 ± 14.1	51.1±15.4	0.157
AVA (cmý)	0.67 ± 0.16	0.68 ± 0.16	0.66±0.16	0.036
Aortic annulus (cm)	2.15 ± 0.20	2.14 ± 0.2	2.15±0.2	0.672
sPAP (mmHg)	44.0 ± 16.9	44.1 ± 17.3	44.0±16.8	0.988
Moderate to severe aortic regurgitation (%)	24 (4.4)	7 (4.3)	17 (4.4)	0.995
Moderate to severe mitral regurgitation (%)	69 (12.7)	18 (11.0)	51 (13.3)	0.6 48
MSCT, annulus (mm)	24.6 ± 2.4	23.1 ± 2.2	24.4±1.5	0.318
MSCT, annulus area (cm2)	481.9 ± 95.9	474.1 ± 89.6	485.2±98.5	0.311
MSCT, annulus perimeter (mm)	77.4 ± 7.5	76.8 ± 7.2	77.6±7.7	0.318
MSCT, mean CFA size (mm)	7.5 ± 1.1	7.2 ± 1.2	7.7±1.1	0.019

AF=atrial fibrillation; ASA=acetylsalicylic acid; AVA=aortic valve area; BMI=body mass index; CABG=coronary artery bypass grafting; CAD=coronary artery disease; CFA=common femoral artery; CK-MB=creatine kinase-myocardial band; COPD=chronic obstructive pulmonary disease; CRP=C-reactive protein; DM=diabetes mellitus; DOAC=direct oral anticoagulant; EuroSCORE=European System for Cardiac Operative Risk Evaluation; HbA1c=hemoglobin A1c; HDL=high-density lipoprotein; HL=hyperlipidemia; HT=hypertension; LA=left atrium; LDL=low-density lipoprotein; LVEDD=left ventricular end-diastolic diameter; LVEF=left ventricular ejection fraction; LVESD=left ventricular end-systolic diameter; MI=myocardial infarction; MSCT=multi-slice computed tomography; NYHA=New York Heart Association; PAD=peripheral artery disease; PCI=percutaneous coronary intervention; sPAP=systolic pulmonary artery pressure; STS=Society of Thoracic Surgeons

**Fig. 1:**
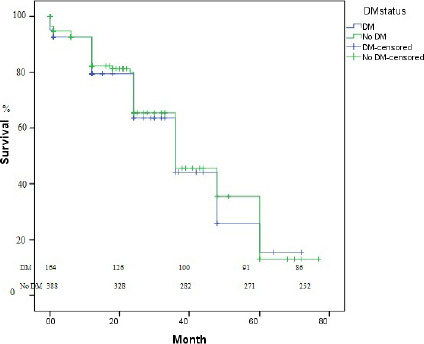
Kaplan–Meier analysis of survival curves in patients with diabetes mellitus (DM) and without DM. Overall survival probability was not significantly different in those patients (DM 38.5 ± 2.7 months; 95% confidence interval [CI] 33.1-43.9; no DM 40.8 ± 2.0 months; 95% CI 36.7-44.9; log-rank P=0.512).

**Fig. 2:**
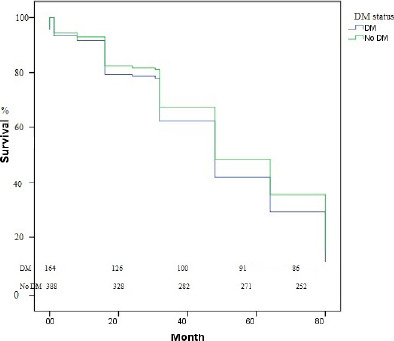
Cox age, body mass index, previous myocardial infarction, previous percutaneous coronary intervention, coronary artery bypass grafting, hypertension, hyperlipidemia history, adjusted regression analysis of survival curves in patients with diabetes mellitus (DM) and without DM. Overall survival probability was not different in those patients (P=0.736; 95% confidence interval 0.889 [0.586-1.349]).

The procedural features were presented in Table 2. They were similar within the two groups with a comparable proportion of the types of transcatheter heart valve (THV), the sizes of THV, access routes, and closure devices used. Device success was 97.0% in the DM group and 95.9% in the no DM group, and there was no statistical difference (*P*=0.543). The in-hospital and postTAVI follow-up outcomes compared among DM and no DM groups were shown in Table 3. The in-hospital mortality was similar between the groups (4.9% *vs.* 3.6%, *P*=0.490). The rates of major or minor vascular results and percutaneous closure device failure were not significantly different between the groups. Although acute kidney injury was observed more frequently in the DM group (2.4% *vs.* 0%, *P*=0.021), no statistical difference was observed between postTAVI chronic kidney stages (*P*=0.181). Similarly, improvement was observed in functional capacity and echocardiographic parameters in both groups during follow-up (Table 4). The systolic pulmonary artery pressure, which was similar before TAVI, was significantly lower in the DM group at 30-day follow-up (34.1 ± 13.4 *vs.* 37.7 ± 13.8 mmHg, *P*=0.037). First-year mortality was 15.0% for patients in DM group and 11.2% for those in the no DM group (*P*=0.282). Kaplan– Meier analysis of survival curves in patients with and without DM was performed. Overall survival probability was not significantly different in those patients (DM 38.5 ± 2.7 months; 95% confidence interval [CI] 33.1-43.9; no DM 40.8±2.0 months; 95% CI 36.7-44.9; log-rank *P*=0.512) (Figure 1). Cox age, body mass index, previous MI, previous PCI, coronary artery bypass grafting, HT, and HL history were included in the adjusted regression analysis of survival curves in DM and no DM groups. Overall survival probability was not different in those patients (*P*=0.736; 95% CI 0.889 [0.586-1.349]) (Figure 2).

**Table 2:** Procedure details, related complications, and outcomes.

Parameters	All	DM	No DM	*P*-value
	n=552	n=164	n=388	
Closure method, n (%)				0.427
Prostar™	179 (34.2)	48 (31.0)	131 (35.6)	
ProGlide™	332 (63.5)	102 (65.8)	230 (62.5)	
Cut-down	12 (2.3)	5 (3.2)	7 (1.9)	
Transaxillary access, n (%)	20 (3.7)	8 (4.9)	12 (3.1)	0.318
Valve size, mm, n (%)				0.838
20	2 (0.4)	-	2 (0.5)	
23	230 (41.7)	73 (44.8)	157 (40.5)	
25	14 (2.5)	4 (2.5)	10 (2.6)	
26	226 (41.0)	65 (39.9)	161 (41.5)	
27	6 (1.1)	1 (0.6)	5 (1.3)	
29	73 (13.2)	20 (12.3)	53 (13.7)	
Edwards SAPIEN XT®, n (%)	475 (86.3)	136 (82.9)	340 (87.7)	0.168
Edwards SAPIEN 3®, n (%)	45 (8.2)	19 (11.6)	26 (6.7)	0.055
LOTUS™, n (%)	24 (4.3)	7 (4.4)	17 (4.3)	0.952
ACURATE Neo™, n (%)	6 (1.1)	1 (0.6)	5 (1.3)	0.412
PostTAVI creatinine (mg/dl)	0.98 ± 0.40	1.04 ± 0.52	0.95 ± 0.33	0.021
PostTAVI CKD, n (%)				0.181
Stage 1	90 (17.3)	33 (21.3)	57 (15.6)	
Stage 2	257 (49.3)	65 (41.9)	192 (52.5)	
Stage 3a	104 (20.0)	30 (19.4)	74 (20.2)	
Stage 3b	52 (10.0)	19 (12.3)	33 (9.0)	
Stage 4	16 (3.1)	7 (4.5)	9 (2.5)	
Stage 5	2 (0.4)	1 (0.6)	1 (0.3)	
PostTAVI hemoglobin (mg/dl)	10.6 ± 1.7	10.6 ± 1.7	10.4 ± 2.1	0.308
PostTAVI troponin (pg/ml)	309.1 ± 812.1	309.1 ± 812.1	212.8 ± 431.0	0.122
PostTAVI CK-MB (ng/ml)	7.5 ± 5.9	7.5 ± 5.9	14.3 ± 98.8	0.591

CK-MB=creatine kinase-myocardial band; CKD=chronic kidney disease; DM=diabetes mellitus

**Table 3:** Follow-up outcomes.

Parameters	All	DM	No DM	*P*-value
	n=552	n=164	n=388	
Device success (%)	530 (96.2)	159 (97.0)	371 (95.9)	0.543
Pacemaker, n (%)	40 (7.3)	9 (5.5)	31 (8.0)	0.462
Stroke, n (%)	4 (0.7)	2 (1.2)	2 (0.5)	0.376
Pericardial effusion, n (%)	10 (1.8)	3 (1.8)	7 (1.9)	0.584
Emerging arrhythmia, n (%)				0.587
AF	20 (3.6)	5 (3.0)	15 (3.9)	
VT	3 (0.5)	1 (0.6)	2 (0.5)	
LBBB	14 (2.5)	6 (3.7)	8 (2.1)	
Major vascular complication, n (%)	37 (6.7)	10 (6.0)	27 (6.9)	0.159
Closure device failure, n (%)	11.0 (2.0)	1 (0.6)	10 (2.6)	0.176
Acute kidney injury, n (%)	4 (0.7)	4 (2.4)	-	0.021
Discharge time (days)	4.5 ± 2.3	4.7 ± 2.5	4.4 ± 2.2	0.151
30-day NYHA, n (%)				0.918
1	139 (41.6)	41 (41.4)	98 (41.7)	
2	171 (51.2)	50 (50.5)	121 (51.5)	
3	24 (7.2)	8 (8.1)	16 (6.8)	
6-month NYHA, n (%)				0.216
1	87 (62.1)	22 (53.7)	65 (65.7)	
2	51 (36.4)	19 (46.3)	32 (32.3)	
3	2 (1.4)	-	2 (2.0)	
1-year NYHA, n (%)				0.140
1	67 (79.8)	14 (66.7)	53 (84.1)	
2	16 (19.0)	7 (33.3)	9 (14.3)	
3	1 (1.2)	-	1 (1.6)	
In-hospital mortality, n (%)	22 (4.0)	8 (4.9)	14 (3.6)	0.490
30-day mortality, n (%)	11 (2.2)	4 (2.7)	7 (2.0)	0.617
6-month mortality, n (%)	7 (1.6)	-	7 (2.3)	0.080
1-year mortality, n (%)	51 (12.3)	19 (15.0)	32 (11.2)	0.282
Total mortality, n (%)	158 (28.7)	52 (31.7)	106 (27.4)	0.306

AF=atrial fibrillation; DM=diabetes mellitus; LBBB=left bundle branch block; NYHA=New York Heart Association; VT=ventricular tachycardia

**Table 4:** Follow-up echocardiographic parameters.

Parameters	All	DM	No DM	*P*-value
	n=552	n=164	n=388	
PostTAVI LVEF (%)	54.1 ± 12.7	52.6 ± 13.8	54.8 ± 12.2	0.076
PostTAVI aortic mean gradient (mm Hg)	10.5 ± 3.9	10.5 ± 3.6	10.3 ± 4.0	0.977
PostTAVI sPAP (mmHg)	36.9 ± 13.3	36.9 ± 13.6	36.9 ± 13.1	0.993
PostTAVI PVL (%)				0.542
Mild	94 (17.9)	27 (17.6)	67 (18.0)	
Moderate	5 (1.0)	-	5 (1.3)	
30-day LVEF (%)	55.2 ± 11.4	54.9 ± 12.6	55.3 ± 10.8	0.768
30-day aortic mean gradient (mm Hg)	11.0 ± 4.4	11.2 ± 3.4	10.9 ± 4.8	0.580
30-day sPAP (mmHg)	37.3 ± 13.0	34.1 ± 13.4	37.7 ± 13.8	0.037
30-day PVL (%)				0.742
Mild	52 (17.2)	13 (14.4)	39 (18.3)	
Moderate	6 (2.0)	1 (1.1)	5 (2.3)	
6-month LVEF (%)	58.0 ± 9.0	56.5 ± 11.6	58.7 ± 7.6	0.195
6-month aortic mean gradient (mm Hg)	11.9 ± 5.1	12.1 ± 5.2	11.8 ± 5.1	0.756
6-month sPAP (mmHg)	37.3 ± 13.0	36.7 ± 14.5	37.5 ± 12.4	0.778
6-month PVL (%)				0.649
Mild	23 (23.7)	8 (29.6)	15 (21.4)	
Moderate	-	-	-	
1-year LVEF (%)	58.5 ± 8.7	56.6 ± 10.4	59.2 ± 7.8	0.201
12.2 ± 4.5	11.0 ± 3.5	12.7 ± 4.8	0.096
1-year sPAP (mmHg)	36.1 ± 14.5	32.3 ± 14.0	37.5 ± 14.5	0.114
1-year PVL (%)				0.857
Mild	29 (22.1)	10 (25.0)	19 (20.9)	
Moderate	6 (0.8)	-	1 (1.1)	

DM=diabetes mellitus; LVEF=left ventricular ejection fraction; PVL=paravalvular leakage; sPAP=systolic pulmonary artery pressure

Two hundred ninety-six patients had HbA1c levels; 93 (31.4%) of them were in the ≥ 6.5 group, and the remaining were in the ≤ 6.49 group. When analyzing outcomes among the HbA1c ≥ 6.5 patients *vs.* HbA1c ≤ 6.49 patients, we found that there was a statistical difference between these groups in total mortality (34.4% *vs.* 15.8%, *P*<0.001, respectively). DM was not an independent predictor of mortality in multivariable logistic regression analysis (hazard ratio [HR] 1.80, 95% CI 0.32-9.97; *P*=0.499). The only independent predictors were Society of Thoracic Surgeons (STS) score (HR 1.28, 95% CI 1.09-1.51; *P*=0.003) and HbA1c level ≥ 6.5 (HR 10.78, 95% CI 2.58-21.50; *P*=0.003).

## DISCUSSION

In this study, we evaluated the impact of DM and HbA1c status on the outcomes and survival after TAVI. The main results of the study are (1) about one-third of the patients who underwent TAVI in our institution had DM; (2) there was no significantly different procedural complications in patients with or without DM; (3) mortality and survival rates were similar in groups with and without DM; (4) HbA1c, an indicator of long-term blood glucose regulation, may be correlated with a higher mortality rate in postTAVI patients; (5) HbA1c was an independent mortality predictor, such as the STS score.

Patients with diabetes are at higher risk when undergoing coronary intervention or cardiac operation^[5,6]^. DM, but not HbA1c, is included in the STS risk score as a poor prognostic predictor after cardiac surgery^[18]^. The reduced wound healing, increased platelet activity, a higher risk for infections, and endothelial dysfunction are major factors that increase the risk of complications in diabetic patients^[19,20]^. Moreover, patients with diabetes are often present with comorbidities such as HT, HL, history of MI, or CAD as in our study, which raises the surgical risk. Severe AS and DM are both common among older patients, and DM was correlated with significantly poorer outcomes after surgical aortic valve replacement (SAVR)^[6]^. TAVI has been shown to serve as a feasible option for inoperable, high-, and intermediate-risk patients. Therefore, a less invasive treatment option like the TAVI procedure in diabetic patients seems to be a good alternative. Although there is no randomized controlled study on this subject, there are retrospective data, observational data, and registry in the literature. The impact of DM on procedural outcomes and survival after TAVI is still controversial. Similar to previous studies, in our real world registry on 552 patients, around 1/3 of the patients undergoing TAVI have DM^[7]^. Puls et al.^[8]^ reported that DM was a significant predictor of short- and long-term mortality after TAVI. We found that the DM was not associated with procedural complications and long-term mortality. In their study, including 300 patients, the majority of TAVI are transapical, unlike our study^[8]^. In this study, the reasons for more mortality and complications are in the DM group; DM patients were at high risk, while no DM group was at intermediate risk according to STS score — the transfemoral method, recommended today, was less used, and mortality (18.3% *vs.* 7.3%) and complication rates were higher because of the use of old technology. Conrotto et al.^[7]^ and Abramowitz et al.^[9]^ presented similar results in two separate studies, that short-term mortality or rates of complications after TAVI were not affected with DM and insulin-treated DM, but not orally treated DM. The effect of DM on patients undergoing valve replacement (TAVI and SAVR) was investigated in the Spanish registry of Mendez-Bailon M et al.^[11]^ They found that DM does not increase in-hospital mortality in patients with AS requiring valvular replacement either through open surgery or transcatheter aortic valve replacement. But this study has a major limitation based on a central database, therefore it lacks some proper clinical parameters such as glycemic control, glycated hemoglobin, treatments during hospitalization, or left ventricular ejection fraction. Tokarek T. et al.^[12]^ showed that there were no significant differences in 30-day and 12-month all-cause mortality among groups and that both DM and no DM groups resemble to have a comparable quality of life outcomes through long-term follow-up. Similarly, in our study, a significant improvement was observed in functional capacity in both groups. More specifically, in a study investigating the effect of vascular complications in TAVI in patients with and without diabetes^[13]^, Lareyre F. et al.^[13]^ presented that the presence of DM did not affect the procedural characteristics and was not associated with poorer 30-day death and vascular complications. According to the findings in the meta-analysis, which included 16 studies and 13,253 patients, DM did not impact 30-day and 1-year all-cause death on patients after TAVI, and DM did not increase the risk of 30-day complications after TAVI^[10]^. However, this meta-analysis had serious limitations such as heterogeneity and publication bias. In addition, HbA1c was not investigated in these studies, and knowledge about its effect on TAVI is more limited than about DM. In our study, it was shown that HbA1c ≥ 6.5 was an independent predictor of mortality. Conrotto et al.^[7]^ evaluated the effect of DM status on the result of TAVI and stratified outcomes, according to the patients’ initial HbA1c levels without medications and history, in other study. Similar to our results, they found that HbA1c level > 6.5 was independently correlated with all-cause mortality compared with HbA1c of < 5.7%, whereas an HbA1c level from 5.7 to 6.49 was not. Possibly, with large, randomized studies to be conducted in the future, it will be recognized that HbA1c should be included in the scoring systems in addition to DM and medication type.

### Limitations

Our study has some limitations of a single-center, retrospective study, and generalization of the outcomes may not be applicable. Glycemic control (HbA1c levels could not be measured for all patients) and term of DM before TAVI were not orderly collected and hence not accessible for investigation. We do not have complete medicine data, which could be the parameter that can affect outcomes. Therefore, a prospective randomized study with more patients, glycemic parameters including fasting glycaemia, HbA1c, or insulin resistance parameters, and longer follow-up time is needed.

## CONCLUSION

We here determine that the TAVI procedure can be performed safely and effectively in patients regardless of their DM status, and DM was not correlated with an elevated mortality risk or complication rates after TAVI. Also, in our study, it was shown that mortality was higher in those with HbA1c ≥ 6.5, and it was an independent predictor for long-term mortality.

## Abbreviations, Acronyms & Symbols

AF: Atrial fibrillation
AS: Aortic stenosis
ASA: Acetylsalicylic acid
AVA: Aortic valve area
BMI: Body mass index
CABG: Coronary artery bypass grafting
CAD: Coronary artery disease
CFA: Common femoral artery
CI: Confidence interval
CKD: Chronic kidney disease
CK-MB: Creatine kinase-myocardial band
COPD: Chronic obstructive pulmonary disease
HT: Hypertension
LA: Left atrium
LBBB: Left bundle branch block
LDL: Low-density lipoprotein
LVEDD: Left ventricular end-diastolic diameter
LVEF: Left ventricular ejection fraction
LVESD: Left ventricular end-systolic diameter
MI: Myocardial infarction
MSCT: Multi-slice computed tomography
NYHA: New York Heart Association
PAD: Peripheral artery disease
PCI: Percutaneous coronary intervention
CRP: C-reactive protein
DM: Diabetes mellitus
DOAC: Direct oral anticoagulant
EuroSCORE: European System for Cardiac Operative Risk Evaluation
HbA1c: Hemoglobin A1c
HDL: High-density lipoprotein
HL: Hyperlipidemia
HR: Hazard ratio
PVL: Paravalvular leakage
SAVR: Surgical aortic valve replacement
sPAP: Systolic pulmonary artery pressure
STS: Society of Thoracic Surgeons
TAVI: Transcatheter aortic valve implantation
THV: Transcatheter heart valve
VARC: Valve Academic Research Consortium
VT: Ventricular tachycardia

## References

[1] Otto CM, Kumbhani DJ, Alexander KP, Calhoon JH, Desai MY, Kaul S (2017). 2017 ACC expert consensus decision pathway for transcatheter
aortic valve replacement in the management of adults with aortic stenosis: a
report of the American college of cardiology task force on clinical expert
consensus documents. J Am Coll Cardiol.

[2] Baumgartner H, Falk V, Bax JJ, De Bonis M, Hamm C, Holm PJ (2017). 2017 ESC/EACTS guidelines for the management of valvular heart
disease. Eur Heart J.

[3] Mack MJ, Leon MB, Thourani VH, Makkar R, Kodali SK, Russo M (2019). Transcatheter aortic-valve replacement with a balloon-expandable
valve in low-risk patients. N Engl J Med.

[4] Popma JJ, Deeb GM, Yakubov SJ, Mumtaz M, Gada H, O'Hair D (2019). Transcatheter aortic-valve replacement with a self-expanding
valve in low-risk patients. N Engl J Med.

[5] Donahoe SM, Stewart GC, McCabe CH, Mohanavelu S, Murphy SA, Cannon CP (2007). Diabetes and mortality following acute coronary
syndromes. JAMA.

[6] Halkos ME, Kilgo P, Lattouf OM, Puskas JD, Cooper WA, Guyton RA (2010). The effect of diabetes mellitus on in-hospital and long-term
outcomes after heart valve operations. Ann Thorac Surg.

[7] Conrotto F, D'Ascenzo F, Giordana F, Salizzoni S, Tamburino C, Tarantini G (2014). Impact of diabetes mellitus on early and midterm outcomes after
transcatheter aortic valve implantation (from a multicenter
registry). Am J Cardiol.

[8] Puls M, Bleckmann A, Jacobshagen C, Danner BC, Hasenfuß G, Seipelt R (2014). Diabetes mellitus erhöht die eingriffsbedingte und die
langfristige Mortalität nach kathetergestützter
Aortenklappenimplantation. Dtsch Med Wochenschr.

[9] Abramowitz Y, Jilaihawi H, Chakravarty T, Mangat G, Maeno Y, Kazuno Y (2016). Impact of diabetes mellitus on outcomes after transcatheter
aortic valve implantation. Am J Cardiol.

[10] Sun Y, Liu X, He Y, Tang M, Zhu Q, Xu X (2017). Meta-analysis of impact of diabetes mellitus on outcomes after
transcatheter aortic valve implantation. Am J Cardiol.

[11] Mendez-Bailon M, Lorenzo-Villalba N, Muñoz-Rivas N, de Miguel-Yanes JM, De Miguel-Diez J, Comín-Colet J (2017). Transcatheter aortic valve implantation and surgical aortic valve
replacement among hospitalized patients with and without type 2 diabetes
mellitus in Spain (2014-2015). Cardiovasc Diabetol.

[12] Tokarek T, Dziewierz A, Wiktorowicz A, Bagienski M, Rzeszutko L, Sorysz D (2018). Effect of diabetes mellitus on clinical outcomes and quality of
life after transcatheter aortic valve implantation for severe aortic valve
stenosis. Hellenic J Cardiol.

[13] Lareyre F, Mialhe C, Bourlon F, Habib Y, Dommerc C, Raffort J (2019). Diabetes mellitus is not associated with worse vascular outcome
following percutaneous transfemoral transcatheter aortic valve
implantation. Acta Cardiol.

[14] Tzamalis P, Herzberger V, Bergmann J, Wuerth A, Bramlage P, Schroefel H (2019). The association of diabetes mellitus treated with oral
antidiabetic drugs and insulin with mortality after transcatheter valve
implantation: a 3-year follow-up of the TAVIK registry. Cardiovasc Diabetol.

[15] Chorin E, Finkelstein A, Banai S, Aviram G, Barkagan M, Barak L (2015). Impact of diabetes mellitus and hemoglobin A1C on outcome after
transcatheter aortic valve implantation. Am J Cardiol.

[16] Kappetein AP, Head SJ, Généreux P, Piazza N, van Mieghem NM, Blackstone EH (2012). Updated standardized endpoint definitions for transcatheter
aortic valve implantation: the valve academic research consortium-2
consensus document. J Am Coll Cardiol.

[17] Duran Karaduman B, Ayhan H, Keleş T, Bozkurt E (2020). Evaluation of procedural and clinical outcomes of transcatheter
aortic valve implantation: a single-center experience. Anatol J Cardiol.

[18] O'Brien SM, Shahian DM, Filardo G, Ferraris VA, Haan CK, Rich JB (2009). The society of thoracic surgeons 2008 cardiac surgery risk
models: part 2--isolated valve surgery. Ann Thorac Surg.

[19] Salomon NW, Page US, Okies JE, Stephens J, Krause AH, Bigelow JC (1983). Diabetes mellitus and coronary artery bypass. Short-term risk and
long-term prognosis. J Thorac Cardiovasc Surg.

[20] Williams SB, Goldfine AB, Timimi FK, Ting HH, Roddy MA, Simonson DC (1998). Acute hyperglycemia attenuates endothelium-dependent vasodilation
in humans in vivo. Circulation.

